# Clinical implementation of next-generation sequencing in tertiary health system: the Rijeka retrospective study

**DOI:** 10.3389/fgene.2026.1812702

**Published:** 2026-09-01

**Authors:** Nina Pereza, Sanja Dević Pavlić, Tea Mladenić, Željka Hrupački, Dorotea Vukelić Drašković, Luca Lovrečić, Aleš Maver, Jadranka Vraneković, Iva Bilić Čače, Igor Prpić, Ivona Butorac Ahel, Vladimira Vuletić, Koraljka Benko, Tea Čaljkušić Mance, Marko Klarić, Nada Starčević Čizmarević, Ivana Babić Božović, Goran Hauser, Alen Ružić, Saša Ostojić, Borut Peterlin

**Affiliations:** 1 Department of Medical Biology and Genetics, Faculty of Medicine, University of Rijeka, Rijeka, Croatia; 2 Clinical Hospital Centre Rijeka, Clinic for Pediatrics, Rijeka, Croatia; 3 Department of Pediatrics, Faculty of Medicine, University of Rijeka, Rijeka, Croatia; 4 Clinical Institute for Genomic Medicine, University Hospital Centre Ljubljana, Ljubljana, Slovenia; 5 Faculty of Medicine, University of Ljubljana, Ljubljana, Slovenia; 6 Clinical Hospital Centre Rijeka, Clinic for Neurology, Rijeka, Croatia; 7 Department of Neurology, Faculty of Medicine, University of Rijeka, Rijeka, Croatia; 8 Clinical Hospital Centre Rijeka, Clinic for Cardiovascular Diseases, Rijeka, Croatia; 9 Department of Internal Medicine, Faculty of Medicine, University of Rijeka, Rijeka, Croatia; 10 Clinical Hospital Centre Rijeka, Clinic for Ophthalmology, Rijeka, Croatia; 11 Department of Ophthalmology, Faculty of Medicine, University of Rijeka, Rijeka, Croatia; 12 Clinical Hospital Centre Rijeka, Clinic for Gynecology and Obstetrics, Rijeka, Croatia; 13 Department of Gynecology and Obstetrics, University of Rijeka, Faculty of Medicine, Rijeka, Croatia; 14 Department of Internal Medicine, Clinical Hospital Centre Rijeka, Rijeka, Croatia

**Keywords:** exome sequencing, genetic testing, genomic medicine, indications, next-generation sequencing

## Abstract

**Objective:**

This study aimed to assess the diagnostic yield, clinical indications, and utility of next-generation sequencing (NGS) testing since its implementation through collaboration between the University of Rijeka Faculty of Medicine and the Clinical Hospital Centre Rijeka.

**Materials and Methods:**

This retrospective study included patients referred between 2018 and 2023 from the Clinical Hospital Centre Rijeka to the University of Rijeka Faculty of Medicine for genetic testing, primarily using exome sequencing.

**Results:**

Between April 2018 and December 2023, 412 patients were referred for exome sequencing, of whom 353 (85.7%) underwent diagnostic genetic testing. A notable increase in tests ordered was observed over time. Patients were most frequently referred from Pediatrics (55.0%), Neurology (29.5%), Cardiology (7.4%), Ophthalmology (3.4%), and others (4.7%). A diagnosis was confirmed in 103/353 patients, corresponding to an overall diagnostic yield of 29.2%, and an adjusted diagnostic yield of 27.2% after collapsing related individuals into single family units. In these confirmed cases, 83 distinct disorders involving 71 unique genes were identified, with most patients showing heterozygous variants and several recurrent disorders and genes. Variants of uncertain significance were reported in 35/353 (9.9%) patients.

**Conclusion:**

The 27.2% diagnostic yield demonstrates effective integration of NGS into tertiary clinical practice. The recent introduction of medical genetics specialization is expected to further improve referral quality, variant interpretation, and overall diagnostic outcomes.

## 1 Introduction

Medical genetics is one of the fastest developing medical specializations and, in recent years, genetic testing has evolved to comprehensive genomic testing, which is no longer used just for scientific research but is also implemented in everyday clinical practice (Collins and Mckusick, 2001; Mathew, 2001; Bell, 1998). Although this intensive and rapid progress has ensured the widespread availability of genetic testing even outside the healthcare system, such intensive growth of the field has opened new important questions, such as the appropriate choice of patients for genetic testing and implementing diagnostic pathways for patient referral of complex aetiologies (Rudolf and Peterlin, 2009; Souche et al., 2022).

Next-generation sequencing (NGS) is increasingly being adopted in genetic testing due to its capacity for rapid and high-throughput genomic analysis. It is applied as a comprehensive diagnostic tool for the detection of genetic disorders, through whole-genome sequencing (WGS), whole-exome sequencing (WES), or targeted gene panels. Additionally, WES/WGS enables the investigation of genes beyond predefined clinical hypotheses, thereby enhancing diagnostic precision and expanding the scope of genetic evaluation (Vinkšel et al., 2021). With its introduction into clinical practice, numerous medical specialties have been transformed by improved understanding of disease mechanisms and the development of personalized therapeutic strategies.

In countries facing a shortage of trained medical geneticists, such as Croatia, non-genetic healthcare professionals play an increasingly important role in the integration of NGS into clinical care. While formal training in medical genetics remains limited, even a basic understanding of genomic testing and its appropriate clinical indications can support timely and accurate patient referrals. Considering the complexity and data-intensive nature of NGS, targeted guidance and accessible educational resources can serve as practical tools to support clinicians in identifying patients who may benefit from testing (Matthijs et al., 2016).

Such measures represent an essential step toward optimizing the clinical utility of genomic diagnostics, particularly in settings where the genetic workforce is still developing. In this context, updated recommendations from EuroGentest and the Horizon 2020 Solve-RD project highlight the importance of establishing clearly defined diagnostic pathways to ensure that genomic testing is applied in the most efficient and clinically relevant manner (Souche et al., 2022). Responding to this need, a structured diagnostic pathway for NGS was implemented in 2018 at the University of Rijeka, Faculty of Medicine, in collaboration with the Clinical Hospital Centre Rijeka.

While the diagnostic performance of exome sequencing has been extensively evaluated across numerous clinical settings, considerably less attention has been devoted to understanding how these technologies are successfully integrated into routine healthcare systems. Beyond diagnostic yield alone, the implementation of NGS requires coordinated clinical referral pathways, multidisciplinary collaboration, standardized variant interpretation, and continuous adaptation of healthcare infrastructure. Reports describing real-world implementation are therefore valuable because they provide practical evidence regarding organizational and referral patterns, and factors influencing diagnostic effectiveness that may inform similar initiatives in other tertiary healthcare centres.

To evaluate the implementation and clinical performance of our genomic diagnostic pathway, this retrospective study assessed the diagnostic yield of NGS testing following its integration into routine tertiary healthcare. In addition to evaluating diagnostic yield, we analyzed referral patterns, clinical indications, and overall testing outcomes to characterize the implementation process and identify practical aspects of integrating genomic diagnostics into routine clinical care. By describing these implementation-related outcomes, the study offers insights that may inform the development and optimization of similar genomic diagnostic services in other tertiary healthcare settings.

## 2 Materials and methods

This retrospective study included 412 patients referred from the Clinical Hospital Centre Rijeka to the University of Rijeka, Faculty of Medicine for exome sequencing between April 2018 and December 2023. Clinical and genetic data were collected from internal laboratory records and patient medical documentation, including demographics, referring department, type of testing performed (diagnostic testing or segregation analysis), clinical indication, and genetic test results. This manuscript includes only patients who underwent WES/WGS following negative first-line testing. Prior testing using standard diagnostic algorithms was performed according to the appropriate diagnostic pathway. Informed consent was obtained from all patients prior to testing. All data were anonymized prior to analysis.

### 2.1 Sequencing

Peripheral blood samples were obtained from all referred patients, and genomic DNA was extracted using the Qiagen FlexiGene DNA Kit (Qiagen GmbH, Hilden, Germany). DNA quality and concentration were assessed using a NanoDrop spectrophotometer (FischerBiotec, France).

All NGS analyses, including WES, were performed in collaboration with the Clinical Institute for Genomic Medicine in Ljubljana using the Illumina NovaSeq 6000 platform (Illumina Inc.). Prior to sequencing, DNA fragmentation and enrichment were performed according to the TwistExomev6_0_CNV protocol. After duplicate removal, reads were aligned to the UCSC hg19 reference genome using the BWA algorithm (v0.6.3), and variant calling was performed using the GATK framework (v2.8). Only variants with a quality score >30 and depth ≥5 were included in downstream analyses. Variant annotation was performed using ANNOVAR and snpEff, with pathogenicity predictions retrieved from the dbNSFP v2 database. Reference gene models and transcript sequences were based on the RefSeq database. Structural variants were assessed using the CONIFER v0.2.2 algorithm. Variants with a population frequency >1% in the 1,000 Genomes and ESP6500 databases, as well as synonymous, intronic (outside canonical splice sites), and non-target-region variants, were filtered out. The bioinformatic analysis followed a modified analytical pipeline of the Center for Mendelian Genomics (version 0.5). Sequencing achieved a median coverage of 177×, with >99.9% of target regions covered at ≥10× depth, enabling >99% sensitivity for homozygous variants and >90% sensitivity for heterozygous variants within targeted regions (Meynert et al., 2014). Confirmation of NGS results, if needed, as well as of inheritance patterns was performed using methods appropriate to the specific variant type (e.g., MLPA, triplet-primed PCR, Sanger sequencing).

Variant classification was performed according to ACMG/AMP variant interpretation guidelines which classifies variants as following: class 1 – benign variants, class 2 – likely benign variants, class 3 – variants of uncertain significance (VUS), class 4 – likely pathogenic variants, class 5 – pathogenic variants (Richards et al., 2015), with modifications in accordance with ACGS recommendations for variant classification and reporting (https://www.acgs.uk.com/quality/best-practice-guidelines/). Where applicable, additional gene- or disease-specific specifications published by ClinGen Variant Curation Expert Panels (VCEPs) and ClinGen Sequence Variant Interpretation (SVI) Working Groups were applied. Strength modifications of individual ACMG criteria were applied according to ACGS and ClinGen SVI recommendations, where applicable. In particular, PM3 evidence weighting for recessive disorders was adjusted according to the ClinGen SVI recommendations for PM3 specification based on cumulative evidence from published observations in trans with pathogenic variants and associated familial data (https://www.clinicalgenome.org/site/assets/files/3717/svi_proposal_for_pm3_criterion_-_version_1.pdf). In accordance with the guidelines, VUS variants were re-evaluated over a two-year period, and variants reported as confirmed *de novo* were classified according to ACMG/AMP criteria (Richards et al., 2015).

### 2.2 Statistics

Data analysis was performed using Microsoft Excel by quantifying positive and negative results, the number of patients referred for genetic testing by specialty, and calculating diagnostic yield. Descriptive statistics, including absolute and relative frequencies, were used to summarize categorical variables and highlight trends within the data.

## 3 Results

### 3.1 Total number of tests

In the period from April 2018 to December 2023, a total of 412 tests were conducted. Of 412 patients diagnostic NGS testing was performed on 353 (85.7%) patients. The remaining 59 (14.3%) were family members of patients in whom a variant had been identified through diagnostic NGS testing, and segregation analysis was performed to verify its mode of inheritance.

### 3.2 Analysis of diagnostic NGS testing results

A total of 353 patients were included in the NGS diagnostic cohort, consisting of 54.1% (N = 191) females and 45.9% (N = 162) males. Within this cohort, 43.1% were adults (N = 152), 55.0% were children (N = 194), and 2.0% (N = 7) were fetal samples. The mean age of pediatric patients was 8 years (range 1–17), while the mean age of adult patients was 49 years (range 18–84).

For diagnostic NGS testing (N = 353), pediatricians referred 194 (55.0%) and neurologists 104 (29.5%) patients since 2018, whereas from the beginning of 2023 cardiologists referred 26 (7.4%) patients, ophthalmologists 12 (3.4%), gynecologists 7 (2.0%) and others 10 (2.7%).

Of the total number of tests performed using exome sequencing, positive results (class 5 – pathogenic variants, class 4 – likely pathogenic variants) were obtained in 109/353 (30.9%) patients. The VUS (class 3) were identified in 35/353 (9.9%) of patients.

In a total of 353 diagnostic NGS tests, the most common indications by specialties were: neuromuscular diseases in pediatrics (31/194, 16.0%); dystonia in neurology (27/104, 27.0%); cardiomyopathies in cardiology (17/26, 65.4%); and optic neuropathies in ophthalmology (8/12, 66.7%) (Table 1).

**TABLE 1:** Diagnostic NGS testing results across selected specialties.

Indications by specialty	Referred patients /N (%)	Confirmed diagnosis /N	Diagnostic yield /%	VUS /N
Pediatrics	194 (55.0)	69	35.6	26
neuromuscular diseases	31	7	22.5	4
*epilepsy*	22	8	36.4	7
*cardiomyopathies*	20	4	20.0	5
*multiple congenital anomalies*	20	9	45.0	2
Neurology	104 (29.5)	16	15.4	6
*dystonia*	27	4	14.8	1
Parkinson’s disease	12	1	8.3	1
ALS	10	1	10.0	0
Cardiology	26 (7.4)	4	15.4	1
*cardiomyopathies*	17	4	23.5	1
hereditary arrhythmias	2	0	0	0
Ophthalmology	12 (3.4)	9	75.0	0
*optic neuropathies*	8	7	87.5	0
Others	17 (4.7)	5	29.4	2
hereditary cancer syndromes	5	1	20.0	0
Total	353	103	29.2*	35

* Adjusted diagnostic yield calculated using index cases only was 27.2%.

The referral diagnosis was confirmed in 103 of 353 patients, resulting in an overall diagnostic yield of 29.2% through the identification of (likely) pathogenic variants. The ratio of referred to confirmed diagnoses is shown in Table 1. The diagnostic yield varies considerably across different specialties (15.4%–35.6%), depending on the reason of referral.

However, because siblings were included in the diagnostic NGS cohort (Supplementary Table 1) and in some cases tested concurrently, a family-based analysis was additionally performed in order to avoid inflation of the diagnostic yield due to related individuals. In this adjusted analysis, only index cases were considered, with affected relatives within the same family collapsed into single family units. Using this approach, the adjusted diagnostic yield was 27.2% (96/353).

#### 3.2.1 Identified (likely) pathogenic variants

Supplementary Table 1 presents the identified (likely) pathogenic variants in 103 patients, comprising 83 unique diagnoses and 71 affected genes. Recurrent findings among unrelated individuals included Marfan syndrome in five patients and PTPN11-associated disorders in four patients, while Rett syndrome, Autosomal dominant long QT syndrome 2, and Occult macular dystrophy were each identified in two patients. Among the 96 identified variants, 20 were novel, accounting for 20.8% of the total (Supplementary Table 1) (Landrum et al., 2014). Correspondingly, most patients were heterozygous (83/103, 80.6%), consistent with a predominantly autosomal dominant inheritance pattern, whereas smaller proportions were homozygous (12/103, 11.7%), compound heterozygous (7/103, 6.8%), or hemizygous (1/103, 0.9%) (Supplementary Table 1).

#### 3.2.2 Identified variants of uncertain significance

VUS (class 3) were identified in 35/353 (9.9%) patients (Supplementary Table 2). A total of 36 VUS variants (with one patient carrying two VUS variants) were detected across 30 unique genes. Most variants were present in the heterozygous state (32/36, 88.9%). During the study period, none of the VUS were reclassified.

### 3.3 Segregation analysis

To determine whether the identified variants in patients were inherited or *de novo*, segregation analysis was performed in 59 patients, consisting of 45.8% (N = 27) females and 54.2% (N = 32) males. Within this cohort, 89.8% were adults (N = 53), 10.2% were children (N = 6). The mean age of pediatric patients was 9 years (range 6–12), while the mean age of adult patients was 41 years (range 20–70). The results of segregation analysis showed that the variants were inherited in 7 patients, either paternally (3/7, 42.9%), or from both mother and father (4/7, 57.1%) (Supplementary Table 1). In 10 patients, a *de novo* origin of the variant was confirmed (Supplementary Table 1).

## 4 Discussion

This retrospective study, conducted by the Faculty of Medicine, University of Rijeka, and the Clinical Hospital Centre Rijeka, evaluated the diagnostic performance of NGS in a tertiary healthcare setting. This study extends beyond reporting diagnostic yield by illustrating the practical implementation of clinical exome sequencing within a tertiary healthcare system during the early phases of genomic medicine integration. Although healthcare systems differ in organization and available resources, many institutions face similar challenges when introducing comprehensive genomic testing into routine practice, including establishing referral pathways, developing multidisciplinary expertise, and integrating laboratory and clinical workflows. Our experience therefore provides a real-world example of how these challenges can be addressed while maintaining diagnostic performance comparable to that reported internationally.

Over 6 years, 353 patients underwent NGS testing, with an overall diagnostic yield of 29.2%, and an adjusted diagnostic yield of 27.2% after collapsing related individuals into single family units. The results of this study also underscore the remarkable genetic and clinical heterogeneity among patients referred for molecular diagnostics. In 103 patients with confirmed diagnoses, 83 distinct disorders and 71 unique genes were identified. While most diagnoses and variants were unique, recurrent diagnoses such as Marfan syndrome (OMIM:154700) and PTPN11-associated disorders (OMIM:163950, 151,100) likely reflect the clinical referral patterns and the broad phenotypic spectrum associated with genes such as FBN1 and PTPN11, rather than population-specific founder effects. The predominance of pathogenic (class 5) and likely pathogenic (class 4) variants, along with a high proportion of heterozygous findings (80.6%), indicates a strong contribution of autosomal dominant inheritance. This aligns with evidence that over half of Mendelian disease genes are associated with dominant conditions and may be influenced by population-specific factors, as consanguinity rates in Croatia are low, potentially reducing the burden of autosomal recessive disease (Zschocke et al., 2023; Kovačić Perica et al., 2023). These findings emphasize the importance of comprehensive genetic testing to capture both rare, novel variants and clinically significant recurrent alterations. Furthermore, the identification of *de novo* variants in 10 patients through segregation analysis highlights the added value of family-based testing in strengthening variant interpretation, refining recurrence risk assessment, and supporting clinical management and genetic counseling.

Most referrals in our cohort were initiated by pediatricians (55.0%) and neurologists (29.5%), with the remaining 15.5% coming from specialties such as cardiology, ophthalmology, gynecology, and gastroenterology. The most common indications for testing were neuromuscular diseases, followed by epilepsy, cardiomyopathies, and multiple congenital anomalies, with referrals originating from pediatric subspecialties such as neurology, cardiology, endocrinology, and intensive care. Diagnostic yield tended to be higher in cases with more specific referral diagnoses, particularly when patients were well-characterized with multiple documented clinical features; however, this relationship varied across disease categories and specialties, while broader or less well-defined indications (e.g., developmental delay or speech and language impairment) were generally associated with lower yields. These observations align with previous studies reporting higher diagnostic rates for precise clinical presentations, highlighting the importance of providing detailed phenotypic information to guide variant interpretation (Lee et al., 2014; Lee et al., 2024; Kumar et al., 2023; Hartman et al., 2019).

In our study, VUS were detected in 9.9% of referred patients. The highest VUS rate was observed in the pediatric group, particularly among patients referred for epilepsy and cardiomyopathies (Table 1; Supplementary Table 2). This is not unexpected, as pediatric referrals often involve limited number of clinical features expressed in early childhood, making precise phenotyping more challenging. Moreover, many previous studies do not consistently report VUS rates. A meta-analysis by Pandey et al. showed that only 25 out of 39 included studies provided VUS data (Pandey et al., 2025). Among those, the reported frequency in WES studies ranged from 1% to 34%, with an estimated average of around 8%. These wide variations likely reflect differences in patient populations, phenotypic specificity, interpretation criteria, and the evolving nature of variant classification over time. Our rate falls within this broad range and aligns closely with the meta-analytic average (Pandey et al., 2025).

Differences in diagnostic yield across specialties were also evident. Ophthalmology showed a higher yield, likely due to the more specific clinical presentations in these patients. In contrast, neurology had a comparatively lower yield, as most referred patients presented with neurodegenerative disorders, which are highly heterogeneous. Despite this, confirming a genetic diagnosis in these cases remains clinically valuable for guiding treatment and avoiding unnecessary testing.

Several implementation-related observations emerging from this study may be relevant beyond our institution. First, the progressive increase in referral volume demonstrates growing clinician acceptance of genomic testing following its introduction into routine practice. Second, the predominance of referrals from pediatric and neurological services reflects the clinical specialties in which exome sequencing currently provides the greatest diagnostic benefit, while simultaneously identifying specialties in which broader awareness and utilization may be warranted. Third, close collaboration between clinicians, molecular geneticists, laboratory specialists, and multidisciplinary case discussions proved essential for appropriate test selection, accurate variant interpretation, and clinically meaningful reporting. These findings support the concept that successful implementation depends not only on sequencing technology itself but also on the organization of integrated clinical and laboratory workflows.

Through close collaboration among the University of Rijeka, Clinical Hospital Centre Rijeka, and the Clinical Institute for Genomic Medicine in Ljubljana, an effective diagnostic workflow has been established, ensuring consistent tertiary-level support. Importantly, our experience demonstrates that successful clinical implementation of NGS is achievable within a healthcare system undergoing progressive expansion of genomic medicine resources. The establishment of institutional collaboration between a university-based molecular genetics laboratory and a tertiary clinical centre enabled the development of local genomic diagnostic capacity without requiring an already mature national genomic infrastructure. Such implementation models may be particularly relevant for healthcare systems with comparable organizational structures or limited genomic resources seeking to expand access to precision diagnostics.

The recent introduction of a formal medical genetics’ specialization is expected to further enhance the quality of patient referrals, variant interpretation, and genetic counselling, and to increase diagnostic yield across specialties. This cohort is highly representative of the Croatian population, providing valuable insight into the implementation of NGS in clinical practice. However, some limitations should be considered, including variability in phenotypic descriptions, the relatively small size of certain phenotypic subgroups, the potential influence of simultaneous analysis of related individuals on the diagnostic yield, and the lack of systematic information on the novelty of variants.

## 5 Conclusion

Our results demonstrate an effective pathway for integrating genomic medicine into tertiary healthcare. With a diagnostic yield of 27.2%, consistent with international experience, this study confirms the clinical utility of NGS. Growing clinician awareness and the gradual shift toward personalised care are evident, and the recent introduction of medical genetics specialization is expected to further enhance referral quality, variant interpretation, and genetic counselling. Continued investment in infrastructure and multidisciplinary collaboration will be key to expanding genomic diagnostics and maximizing their impact across all specialties. Beyond demonstrating a diagnostic yield comparable to international experience, this study highlights practical aspects of integrating exome sequencing into routine tertiary care, including referral development, multidisciplinary collaboration, and progressive clinical adoption. These implementation-related findings provide a real-world framework that may assist other healthcare systems seeking to establish or expand clinical genomic services.

## Data Availability

The original contributions presented in this study are publicly available in the ClinVar database. The sequence variants generated and submitted as part of this study, as well as the publicly available ClinVar data analyzed in this study, are described in the article and Supplementary Material, including the relevant accession numbers.
