# Supplementary material for: Clinical implementation of next-generation sequencing in tertiary health system: the Rijeka retrospective study
Source: Front Genet. 2026 Sep 1;17:1812702. doi: 10.3389/fgene.2026.1812702 (PMC13574805; doi:10.3389/fgene.2026.1812702)
Supplement: Supplementary file 1 [file Table1.docx]

Supplementary Material

Supplementary Table 1. Identified (likely) pathogenic variants

| **Patient** | **Referral** | **Diagnosis** | **Gene** | **Transcript Change** | **Protein Change** | **Variant Classification** | **ACMG/AMP criteria** | **Zygosity** | **Novel variant(1)**  **(+/-)** | **ClinVar**  **ID** |
| --- | --- | --- | --- | --- | --- | --- | --- | --- | --- | --- |
| P1 | Pediatric Neurology | Dystrophy type 2D (OMIM: 608099) | *SGCA* | NM_000023.2: c.409G>A | p.Glu137Lys | class 4 | PS1, PM1, PM2, PP3 | homozygote | - | 286049 |
| P2 | Neurology | CADASIL (OMIM: 125310) | *NOTCH3* | NM_000435.2: c.328C>T | p.Arg110Cys | class 5 | PS1, PM1, PM2, PP4, PP3, PP1-M | heterozygote | - | 447831 |
| P3 | Pediatric Emergency and Intensive Care Medicine | Cornelia de Lange syndrome (OMIM: 122470) | *NIPBL* | NM_133433.3: c.2126_2128delinsCTCTCAA | p.Ser709fs | class 4 | PVS1, PM2 | heterozygote^*^ | + | 931097 |
| P4 | Pediatric Cardiology | PTPN11-associated disorder (OMIM:163950, 151100) | *PTPN11* | NM_002834.3: c.922A>G | p.Asn308Asp | class 5 | PS1, PS3, PP2, PP3 | heterozygote | - | 13326 |
| P5 | Neurology | Spastic paraplegia-15 (OMIM: 270700) | *ZFYVE26* | NM_01534.3: c.6278dupC/c.4153C>T | p.Phe2094fs/p.Gln1385Ter | class 4/class 4 | PVS1, PM2/PVS1, PM2 | compound heterozygote^a^ | +/+ | 930779/ 930780 |
| P6 | Pediatric Cardiology | Autosomal dominant non-syndromic supravalvular aortic stenosis (OMIM:185500) | *ELN* | NM_001278939.1: c.1104_1105delGT | p.Val368fs | class 5 | PVS, PM2, PP4 | heterozygote | + | 930778 |
| P7 | Pediatric Cardiology | Autosomal dominant long QT syndrome 2 (OMIM:613688) | *KCNH2* | NM_000238.3: c.1838C>T | p.Thr613Met | class 5 | PS1, PM1, PM2, PP1, PP3 | heterozygote^#^ | - | 67292 |
| P8 | Pediatric Cardiology | Autosomal dominant long QT syndrome 2 (OMIM:613688) | *KCNH2* | NM_000238.3: c.1838C>T | p.Thr613Met | class 5 | PS1, PM1, PM2, PP1, PP3 | heterozygote^#^ | - | 67292 |
| P9 | Pediatric Cardiology | Autosomal dominant long QT syndrome 2 (OMIM:613688) | *KCNH2* | NM_000238.3: c.1838C>T | p.Thr613Met | class 5 | PS1, PM1, PM2, PP1, PP3 | heterozygote | - | 67292 |
| P10 | Ophthalmology | Bosch-Boonstra-Schaaf optic atrophy syndrome (OMIM:615722) | *NR2F1* | NM_005654.6: c.169C>T | p.Gln57Ter | class 5 | PVS1, PS2, PM2 | heterozygote^#^ | + | 931846 |
| P11 | Ophthalmology | Bosch-Boonstra-Schaaf optic atrophy syndrome (OMIM:615722) | *NR2F1* | NM_005654.6: c.169C>T | p.Gln57Ter | class 5 | PVS1, PS2, PM2 | heterozygote^#^ | + | 931846 |
| P12 | Ophthalmology | Retinitis pigmentosa tip 39 (OMIM: 613809) | *USH2A* | NM_206933.2: c.2276G>T/c.13335_13347delinsCTTG | p.Cys759Phe/p.Glu4445_Ser4449delinsAspLeu | class 5/class 5 | PM3_VSTR, PP1_Mod, PP3/PM2, PM3_STR, PM4, PP3 | compound heterozygote | - | 2356/ 438013 |
| P13 | Pediatric Cardiology | Marfan syndrome (OMIM:154700) | *FBN1* | NM_000138.4: c.6658C>T | p.Arg220Ter | class 5 | PVS1, PS4_STR, PM2, PP4 | heterozygote | - | 42407 |
| P14 | Neurology | Recessive form of ataxia-telangiectasia (OMIM: 208900) | *ATM* | NM_000051.3: c.1564_1565delGA/c.8147T>C | p.Glu522fs/p.Val2716Ala | class 5/class 5 | PVS1, PM3_STR, PM2/PS3, PMR_STR, PM2, PM5, PP1, PP3 | compound heterozygote^b^ | - | 127340/ 142700 |
| P15 | Neurology | Neurodegeneration with iron accumulation in the brain 4 (OMIM: 614298) | *C19orf12* | NM_001031726.3: c.204_214del | p.Gly69fs | class 5 | PVS1, PM2, PM3, PP4 | homozygote | - | 31155 |
| P16 | Pediatric pulmonology | Isolated sulfite oxidase deficiency (OMIM: 272300) | *SUOX* | NM_000456.3: c.650G>A | p.Arg217Gln | class 4 | PS3_MOD, PM2, PM3, PM5 | homozygote | - | 3820 |
| P17 | Neurology | Autosomal dominant intellectual developmental disorder-42 (OMIM:616973) | *GNB1* | NM_001282539.1: c.352G>C | p.Asp118.His | class 4 | PM2, PM5, PP2, PP3 | heterozygote | + | 4819630 |
| P18 | Ophthalmology | Autosomal recessive Leber-like hereditary optic neuropathy-1 (OMIM:619382) | *DNAJC30* | NM_032317.2: c.152A>G | p.Tyr51Cys | class 4 | PS3_SUP, PS4, PM2, PP4 | heterozygote | - | 976691 |
| P19 | Neurology | Gabriele-de Vries syndrome (OMIM:617557) | *YY1* | NM_003403.5: c.1123C>T | p.Arg375Ter | class 4 | PVS1_STR, PM2 | heterozygote | + | 3340625 |
| P20 | Pediatric gastroenterology | Autosomal recessive progressive familial intrahepatic cholestasis-3 (OMIM: 602347) | *ABCB4* | NM_018849.3: c.959G>T | p.Ser320Phe | class 5 | PS3, PM3_STR, PM2 | heterozygote | - | 13690 |
| P21 | Pediatric Emergency and Intensive Care Medicine | Currarino syndrome 1 (OMIM: 176450) | *MNX1* | NM_005515.3: c.147_148dupGG | p.Ala50Glyfster173 | class 5 | PVS1, PM2, PP4 | heterozygote | + | 4871885 |
| P22 | Pediatric Cardiology | Brugada syndrome type 1 (OMIM: 601144) | *SCN5A* | NM_001099404.1: c.3673G>A | p.Glu1225Lys | class 4 | PS3, PM2, PS4_SUP, PP3, PP1_SUP | heterozygote | - | 67810 |
| P23 | Pediatric pulmonology | Congenital deficiency of plasminogen type I (OMIM:217090) | *PLG* | NM_000301.3: c.1877+1G>T | splice site | class 4 | PVS1_MOD, PM2, PP4_MOD | homozygote | + | 4871896 |
| P24 | Pediatric Emergency and Intensive Care Medicine | Myastenic syndrome, congental-10 (OMIM: 254300) | *DOK7* | NM_001301072.2:  c.496G>A | p.Gly166Arg | class 4 | PS3_MOD, PM3_STR, PM2 | heterozygote | - | 942353 |
| P25 | Pediatric endocrinology | Combined pituitary hormone deficiency-2 (OMIM:262600) | *PROP1* | NM_006261.4: c.301_302delAG | p.Leu102CysfsTer8 | class 5 | PVS1, PM2, PM3_STR, PS3, PP1_SUP | homozygote^#^ | - | 8098 |
| P26 | Pediatric endocrinology | Combined pituitary hormone deficiency-2 (OMIM:262600) | *PROP1* | NM_006261.4: c.301_302delAG | p.Leu102CysfsTer8 | class 5 | PVS1, PM2, PM3_STR, PS3, PP1_SUP | homozygote^#^ | - | 8098 |
| P27 | Interna-gastro | Lynch syndrome, type I (OMIM: 120435) | *MSH2* | NM_000251.3: c.2021G>A | p.Gly674Asp | class 5 | PS3, PS4, PM2, PM5 | heterozygote | - | 90861 |
| P28 | Neurology | Huntington disease (OMIM:603218) | *HTT* | NM_001388492.1: 20±1 and 42±1 CAG | - | class 5 | - | heterozygote | - | - |
| P29 | Pediatric oncology and hematology | Congenital Thrombocytopenia (PMID:36880038) | *RUNX1* | NM_001754.4: c.1160delG | p.Gly387AlafsTer207 | class 4 | PVS1_STR, PM2 | heterozygote | + | 4871904 |
| P30 | Neurology | Autosomal recessive congenital myotonias (OMIM: 255700) | *CLCN1* | NM_000053.3: c.2680C>T | p.Arg894Ter | class 5 | PVS1, PS3, PM3_STR, PP4 | homozygote | - | 17545 |
| P31 | Neurology | Charcot-Marie-Tooth disease type 2T (OMIM:617017) | *MME* | NM_000902.4: c.467delC | p.Pro156fs | class 5 | PVS1, PM2, PS4_STR | homozygote^#^ | - | 426945 |
| P32 | Neurology | Charcot-Marie-Tooth disease type 2T (OMIM:617017) | *MME* | NM_000902.4: c.467delC | p.Pro156fs | class 5 | PVS1, PM2, PS4_STR | homozygote^#^ | - | 426945 |
| P33 | Pediatric Cardiology | Marfan syndrome (OMIM:154700) | *FBN1* | NM_000138.4: c.3338-2A>G | splice site | class 4 | PVS1_STR, PM2, PP4 | heterozygote | + | 1325493 |
| P34 | Pediatric endocrinology | Hypochondroplasia (OMIM: 146000) | *FGFR3* | NM_001163213.1:  c.1626C>G | p.Asn542Lys | class 5 | PS4_VSTR, PS1, PM2, PM5, PS3 | heterozygote | - | 16338 |
| P35 | Pediatric Neurology | Autosomal dominant Arboleda Tham syndrome (OMIM:616268) | *KAT6A* | NM_006766.5: c.4645G>A | p.Gly1549Ser | class 4 | PS4_MOD, PM1, PM2, PM6, PP3 | heterozygote^*^ | - | 419627 |
| P36 | Pediatric Emergency and Intensive Care Medicine | X-linked lissencephaly type 2 (OMIM: 300215) | *ARX* | NM_139058.3: c.1497delG | p.Leu500fs | class 4 | PS1_STR, PM2, PP4 | hemizygote | + | 2098307 |
| P37 | Pediatric Emergency and Intensive Care Medicine | 3M syndrome-1 (OMIM:273750) | *CUL7* | NM_014780.5: c.920_929delTGGTGCAAGC | p.Leu307fs | class 5 | PVS1, PM2, PM3 | heterozygote | - | 3367199 |
| P38 | Endocrinology and diabetology | Achondroplasia (OMIM:100800) | *FGFR3* | NM_001163213.1: c.1138G>A | p.Gly380Arg | class 5 | PS1, PS3, PS4_STR, PM2,  PM5 | heterozygote | - | 16327 |
| P39 | Pediatric Neurology | Autosomal dominant intellectual developmental disorder-7 (OMIM:614104) | *DYRK1A* | NM_001396.4: c.691C>T | p.Arg231Ter | class 5 | PVS1, PS4_MOD, PM6 | heterozygote^*^ | - | 423502 |
| P40 | Pediatric Neurology | Impaired intellectual development and distinctive facial features with or without cardiac defects (OMIM:616789) | *MED13L* | NM_015335.4: c.2605C>A | p.Pro869Thr | class 4 | PS4_SUP, PM2, PP3, PM5 | heterozygote^*^ | - | 391487 |
| P41 | Pediatric Cardiology | PTPN11-associated disorder (OMIM:163950, 151100) | *PTPN11* | NM_002834.5: c.922A>G | p.Asn308Asp | class 5 | PS4, PM2, PP1, PP2, PP3, PS3 | heterozygote | - | 13326 |
| P42 | Pediatric Neurology | Autosomal recessive axonal Charcot-Marie-Tooth neuropathy type 2EE (OMIM:618400). | *MPV17* | NM_002437.5: c.414G>A/c.122G>A | p.Trp138Ter/p.Arg41Gln | class 4/class 5 | PVS1_STR, PM2/PM2, PM3_STR, PS3 | compound heterozygote | - | 4057910/ 626263 |
| P43 | Pediatric Neurology | Snijders Blok type of X-linked dominant intellectual developmental disorder (OMIM:300958) | *DDX3X* | NM_001356.4: c.1432_1433delAG | p.Arg478fs | class 4 | PVS1, PM2 | heterozygote | + | 2124062 |
| P44 | Pediatric oncology and hematology | Bleeding disorder, platelet-type 15 (OMIM:615193) | *ACTN1* | NM_001130004.1: c.959G>A | p.Arg320Gln | class 4 | PS4_SUP, PS3_MOD, PM2, PP2 | heterozygote | - | 986185 |
| P45 | Pediatric endocrinology | PTPN11-associated disorder (OMIM:163950, 151100) | *PTPN11* | NM_002834.5: c.1510A>G | p.Met504Val | class 5 | PS4_MOD, PS3, PP2, PM6_STR | heterozygote | - | 40562 |
| P46 | Pediatric Neurology | PIK3CA-associated disorder (GeneReviews: NBK153722) | *PIK3CA* | NM_006218.4: c.2740G>A | p.Gly914Arg | class 5 | PP2, PM1_SUP, PM2, PS4, PS2 | heterozygote (mosaic variant 30.1%) | - | 39703 |
| P47 | Pediatric Cardiology | Marfan syndrome (OMIM:154700) | *FBN1* | NM_000138.4: c.4096G>A | p.Glu1366Lys | class 5 | PS4_STR, PM2, PM6, PP4, PP2, PP3 | heterozygote | - | 200036 |
| P48 | Pediatric Cardiology | Central hypoventilation syndrome, congenital with or without Hirschsprung disease (OMIM:209880) | *PHOX2B* | NM_003924.4: c.765_779dup | p.Ala256_Ala260dup | class 5 | PM4, PS4, PS2, PM1, PM2 | heterozygote | - | 429242 |
| P49 | Pediatric Neurology | Autosomal dominant Basal ganglia calcification, type 1 (OMIM:213600) | *SLC20A2* | NM_001257180.2:  c.99delT | p.Phe33fs | class 5 | PVS1, PS4_SUP, PM2, PP4 | heterozygote | - | 930597 |
| P50 | Pediatric Neurology | The spectrum of KMT2B-associated disorders (OMIM:617284, 619934) | *KMT2B* | NM_014727.2: c.4789C>T | p.Arg1597Trp | class 4 | PM2, PS2_MOD, PS4_MOD | heterozygote | - | 1013155 |
| P51 | Pediatric endocrinology | FGFR3-associated skeletal dysplasia with short limbs | *FGFR3* | NM_001163213.1: c.1626C>A | p.Asn542Lys | class 5 | PS1, PS3, PS4_VSTR, PM2, PM5, PP4 | heterozygote | - | 16337 |
| P52 | Neurology | Autosomal dominant hereditary spastic paraplegia type 4 (OMIM: 182601) | *SPAST* | NM_014946.3: c.977delA | p.Asn326fs | class 4 | PVS1, PM2 | heterozygote | - | 834811 |
| P53 | Pediatric endocrinology | Infantile hypercalcemia-1 (OMIM:143880) | *CYP24A1* | NM_000782.5: c.443T>C/c.428_430delAAG | p.Leu148Pro/Glu143del | class 5/class 5 | PS3, PM3_STR, PM2, PM6_SUP, PP4/PS3_SUP, PM3_VSTR, PM4, PP4 | compound heterozygous^c,#^ | - | 631878/ 29677 |
| P54 | Pediatric endocrinology | Infantile hypercalcemia-1 (OMIM:143880) | *CYP24A1* | NM_000782.5: c.443T>C/c.428_430delAAG | p.Leu148Pro/Glu143del | class 5/class 5 | PS3, PM3_STR, PM2, PM6_SUP, PP4/PS3_SUP, PM3_VSTR, PM4, PP4 | compound heterozygous^c,#^ | - | 631878/ 29677 |
| P55 | Urology | BRCA1-associated hereditary breast and ovarian cancer (OMIM:604370) | *BRCA1* | NM_007294.4: c.5266dupC | p.Gln1756fs | class 5 | PVS1, PS4_VSTR | heterozygote | - | 17677 |
| P56 | Pediatric Emergency and Intensive Care Medicine | Primary aldosteronism, seizures, and neurologic abnormalities (OMIM:615474) | *CACNA1D* | NM_000720.4: c.2018T>C | p.Leu673Pro | class 4 | PS2, PM2, PP3, PP2 | heterozygote^*^ | + | 1698702 |
| P57 | Neurology | Von Hippel-Lindau syndrome (OMIM: 193300) | *VHL* | NM_000551.3: c.463+2T>C | splice site | class 5 | PVS1, PS4, PM2, PS1_SUP | heterozygote^d^ | - | 569414 |
| P58 | Pediatric Neurology | PIK3CA-spectrum disorders, presenting with macrocephaly and overgrowth (GeneReviews: NBK153722). | *PIK3CA* | NM_006218.4: c.323G>A | p.Arg108His | class 4 | PS3_MOD, PM1, PM2, PP3 | heterozygote (mosaic variant 29.3%)^*^ | - | 280875 |
| P59 | Pediatric nephrology | Polycystic kidney disease, type 2 (OMIM: 613095) | *PKD2* | NM_000297.4: c.710-2A>G | splice site | class 5 | PVS1, PM2, PS4_MOD, PP4 | heterozygote | - | 1285124 |
| P60 | Pediatric Neurology | Myotonia congenita Becker disease  (OMIM: 255700) | *CLCN1* | NM_000083.3: c.501C>G | p.Phe167Leu | class 5 | PM1, PM3_VSTR, PP1_SUP, PP4 | heterozygous | - | 209138 |
| P61 | Pediatric endocrinology | Noonan syndrome 7 (OMIM: 613706) | *BRAF* | NM_004333.6: c.1455G>C | p.Leu485Phe | class 5 | PS4_VSTR, PM2, PP2, PS3 | heterozygote | - | 13975 |
| P62 | Ophthalmology | Optic atrophy 1 (OMIM: 165500) | *OPA1* | NM_130837.2: c.2873_2876delTTAG | p.Val958fs | class 5 | PM2, PM3_VSTR, PM5, PP3 | heterozygote | - | 5082 |
| P63 | Ophthalmology | Wolfram syndrome 1 (OMIM: 222300) | *WFS1* | NM_006005.3: c.1673G>A | p.Arg558His | class 5 | PM2, PM3_VSTR,PM5,PP3 | homozygote | - | 427051 |
| P64 | Pediatric Neurology | Brain small vessel disease 2 (OMIM: 614483) | *COL4A2* | NM_001846.4: c.2312G>T | p.Gly771Val | class 4 | PM1_STR, PM2, PP3 | heterozygote^#^ | + | 4871905 |
| P65 | Pediatric Neurology | Brain small vessel disease 2 (OMIM: 614483) | *COL4A2* | NM_001846.4: c.2312G>T | p.Gly771Val | class 4 | PM1_STR, PM2, PP3 | heterozygote^#^ | + | 4871905 |
| P66 | Fetal medicine and obstetrics | BRAF-associated RASopathy (Noonan syndrome 7, OMIM: 613706) | *BRAF* | NM_004333.6: c.1606G>A | p.Gly469Glu | class 5 | PS2_MOD, PS4, PM1, PM2, PM5, PM6 | heterozygote | - | 13974 |
| P67 | Pediatric Neurology | Cardiofaciocutaneous syndrome (OMIM 115150) | *BRAF* | NM_004333.6: c.1617A>C | p.Lys539Asn | class 5 | PS2_MOD, PS4, PM1, PM2, PM5, PM6 | heterozygote | - | 40371 |
| P68 | Neurology | Amyotrophic lateral sclerosis 1 (OMIM 105400) | *SOD1* | NM_000454.1: c.435G>C | p.Leu145Phe | class 5 | PS4, PS3, PM2, PM5, PP1_STR | heterozygote | - | 586637 |
| P69 | Pediatric Cardiology | Immunodeficiency 14A (OMIM: 615513) | *PIK3CD* | NM_005026.5: c.3061G>A | p.Glu1021Lys | class 5 | PS2, PS3, PS4, PM1, PM2, PP1 | heterozygote | - | 88675 |
| P70 | Pediatric Neurology | KCNQ2-associated Developmental and epileptic encephalopathy, type 7 (OMIM: 613720, 121200) | *KCNQ2* | NM_172107.4: c.637C>T | p.Arg213Trp | class 5 | PS2, PS3, PS4, PM1, PM2, PM5 | heterozygote | - | 21795 |
| P71 | Pediatric Cardiology | Left ventricular noncompaction hypertrophic cardiomyopathy (OMIM:613426) | *MYH7* | NM_000257.4: c.1248T>G | p.Asn416Lys | class 4 | PM1, PM2, PS4_SUP, PS1_SUP | heterozygote | - | 4871906 |
| P72 | Pediatric gastroenterology | PTPN11-associated disorder (OMIM:163950, 151100) | *PTPN11* | NM_002834.5: c.417G>C | p.Glu139Asp | class 5 | PS1_SUP, PS3, PS4, PM2, PM5_SUP, PM6, PP2, PP4 | heterozygote^*^ | - | 40513 |
| P73 | Pediatric Neurology | Autosomal dominant intellectual developmental disorder-42 (OMIM:616973) | *GNB1* | NM_001282539.1:  c.346G>A | p.Gly116Ser | class 4 | PS4_SUP, PM1, PM2, PM5_SUP, PP3 | heterozygote^*^ | - | 444149 |
| P74 | Ophthalmology | Lamb-Shaffer syndrome (OMIM:616803) | *SOX5* | NM_006940.6: c.751C>T | p.Gln251Ter | class 4 | PVS1, PM2 | heterozygote | + | 4871907 |
| P75 | Neurology | Rubinstein-Taybi syndrome, type 2 (OMIM:613684) | *EP300* | NM_001429.4: c.4508dupA | p.Tyr1503Ter | class 4 | PVS1, PM2 | heterozygote | + | 4871908 |
| P76 | Pediatric Emergency and Intensive Care Medicine | Autoimmune polyglandular syndrome type I (OMIM:240300) | *AIRE* | NM_000383.4: c.769C>T/c.151_152dupGA | p.Arg257Ter/p.Glu53fs | class 4 | PVS1, PM3_VSTR, PS3/PVS1, PM2 | compound heterozygote^e^ | - | 3307/? |
| P77 | Pediatric Neurology | Developmental and epileptic encephalopathy-2 (OMIM:300672) | *CDKL5* | NM_001037343.1: c.917dupC | p.Pro307fs | class 4 | PVS1, PM2 | heterozygote^*^ | + | 4871909 |
| P78 | Neurology | Congenital myopathy-16 with tremor (OMIM:618524) | *MYBPC1* | NM_001254718.1:  c.713T>G | p.Leu238Arg | class 5 | PS4_VSTR, PS3_MOD, PM2 | heterozygote | - | 635215 |
| P79 | Pediatric Cardiology | Marfan syndrome (OMIM:154700) | *FBN1* | NM_000138.4: c.6448C>T | p.Arg2150Cys | class 4 | PS4_MOD, PM2, PP2, PP3, PP4 | heterozygote | - | 200084 |
| P80 | Pediatric Cardiology | Familial arrhythmogenic right ventricular dysplasia-9 (OMIM:6090409 | *PKP2* | NM_004572.3: c.368G>A | p.Trp128Ter | class 5 | PVS1, PS4_VSTR, PM2, PP1 | heterozygote | - | 196395 |
| P81 | Pediatric Emergency and Intensive Care Medicine | Marfan syndrome (OMIM:154700) | *FBN1* | NM_000138.4: c.1766A>G | p.Asn589Ser | class 4 | PS4_MOD, PM1, PM2, PM5, PP3 | heterozygote^*^ | - | 549041 |
| P82 | Cardiology | Left ventricular noncompaction and dilatation (OMIM:601494) | *TNNT2* | NM_001276345.2: c.445C>T | p.Arg149Cys | class 4 | PS4, PM2, PP3, PM5 | heterozygote | - | 43640 |
| P83 | Pediatric Neurology | Autosomal recessive limb-girdle muscular dystrophy-1 (OMIM:253600) | *CAPN3* | NM_000070.3: c.550delA | p.Thr184fs | class 5 | PVS1, PS3_SUP, PM3_VSTR, PM2 | heterozygote | - | 17621 |
| P84 | Pediatric Cardiology | Long QT syndrome-3 (OMIM:603830) | *SCN5A* | NM_001099404.1: c.4850_4852delTCT | p.Phe1617del | class 5 | PS4_STR, PS3, PP1_STR, PM2_SUP | heterozygote | - | 201572 |
| P85 | Pediatric Neurology | Parietal foramina-2 (OMIM:609597), Leber's hereditary optic neuropathy (PMID:17254817) | *ALX4, MT-ND4* | NM_021926.4: c.867T>A/  NM_012920.1: c.1019G<A | p.Tyr289Ter/  p.Arg340His | class 4/class 4 | PVS1_STR, PM2/PS4, PS3_MOD, PP1_MOD | heterozygote/  homoplasmic | - | 4871910/9708 |
| P86 | Pediatric Neurology | Rett syndrome (OMIM:312750) | *MECP2* | NM_001110792.2: c.509C>T | p.Thr170Met | class 5 | PS4_VSTR, PS3_SUP, PM1, PM2, PP3 | heterozygote | - | 11811 |
| P87 | Fetal medicine and obstetrics | Osteogenesis imperfecta, type II (OMIM:166210) | *COL1A2* | NM_000089.4: c.3097G>C | p.Gly1033Arg | class 4 | PM1_STR, PM2, PP3 | heterozygote | + | 4871886 |
| P88 | Pediatric Neurology | Rett syndrome (OMIM:312750) | *MECP2* | NM_001110792.2: c.961C>T | p.Arg321Trp | class 5 | PS4, PM1, PM2, PS2_MOD | heterozygote | - | 143749 |
| P89 | Neurology | Idiopathic generalized epilepsy-12 (OMIM:614847) | *SLC2A1* | NM_006516.4: c.694C>T | p.Arg232Cys | class 5 | PS3_SUP, PS4_MOD, PM2, PM5, PP1_STR, PP2 | heterozygote^f^ | - | 37301 |
| P90 | Cardiology | Dilated cardiomyopathy-1EE (OMIM:613252) | *MYH7* | NM_000257.4: c.754T>C | p.Phe252Leu | class 4 | PM2, PM1, PP3, PM5, PS4_SUP | heterozygote | - | 1759485 |
| P91 | Pediatric Cardiology | Left ventricular noncompaction, hypertrophic cardiomyopathy (OMIM: 601494) | *TTN* | NM_001267550.2: c.87341_87343dupGAT | p.Arg29114_Tyr29115insTer | class 4 | PVS1, PM2 | heterozygote | + | 4871887 |
| P92 | Pediatric Neurology | Sotos syndrome (OMIM:117550) | *NSD1* | NM_022455.4: c.3214C>T | p.Arg1702Ter | class 5 | PVS1, PS4_MOD, PM2, PM6 | heterozygote | - | 159303 |
| P93 | Cardiology | LMNA-associated dilated cardiomyopathy type 1A (OMIM:115200) | *LMNA* | NM_170707.4: c.488AUG | p.Thr15Ala | class 4 | PS4_MOD, PM2, PM5 | heterozygote | - | - |
| P94 | Pediatric endocrinology | Autoimmune polyglandular syndrome, type I (OMIM:240300) | *AIRE* | NM_000383.4: c.769C>T | p.Arg257Ter | class 5 | PVS1, PM3_STR, PS3, PP4 | homozygote^g^ | - | 3307 |
| P95 | Pediatric endocrinology | Combined pituitary hormone deficiency-2 (OMIM: 262600) | *PROP1* | NM_006261.4: c.150delA | p.Arg53fs | class 5 | PVS1, PM2, PM3_STR | homozygote | - | 8102 |
| P96 | Pediatric Cardiology | Dilated cardiomyopathy type 1G (OMIM:604145) | *TTN* | NM_001267550.2: c.12478delA | p.Thr4160fs | class 5 | PVS1, PM2, PS4_SUP | heterozygote | - | 1403910 |
| P97 | Cardiology | MYH7-associated cardiomyopathy (OMIM: 613426) | *MYH7* | NM_000257.4: c.1405G>T | p.Asp469Tyr | class 4 | PM1, PM2, PP3, PS4_SUP | heterozygote | - | 42844 |
| P98 | Ophthalmology | Occult macular dystrophy (OMIM:613587) | *RP1L1* | NM_178857.6: c.133C>T | p.Arg45Trp | class 5 | PS4, PP1_STR, PP3 | heterozygote^#^ | - | 2193 |
| P99 | Ophthalmology | Occult macular dystrophy (OMIM:613587) | *RP1L1* | NM_178857.6: c.133C>T | p.Arg45Trp | class 5 | PS4, PP1_STR, PP3 | heterozygote^#^ | - | 2193 |
| P100 | Pediatric Neurology | Benign familial infantile seizures-2 (OMIM:605751) | *PRRT2* | NM_001256442.2: c.649dupC | p.Arg217fs | class 5 | PVS1, PS3, PS4, PP1_STR | heterozygote | - | 65758 |
| P101 | Pediatric Pulmonology | 16p13.11 microdeletion syndrome (ORPHA: 261236) | - | chr16p13.11 | copy number loss | class 5 | 2A | heterozygote | - | - |
| P102 | Pediatric Endocrinology | 18q deletion syndrome (OMIM:601808) | - | chr18q22.1q23 | copy number loss | class 5 | 1A, 3C, 4C | heterozygote | - | - |
| P103 | Pediatric Neurology | Intellectual developmental disorder (OMIM: 618009, 618050, 618906) | - | chr.17q23.2q23.3 | copy number loss | class 5 | 1A, 2A | heterozygote | - | - |

^*^ de novo; ^#^ sibling; ^$^ (+) novel variant, (-) previously reported variant

^a^ pat(c.4153C>T)/mat (c.6278dupC); ^b^ pat(c.8147T>C)/mat(c.1564_1565delGA); ^c^ pat(c.443T>C/c.428_430delAAG); ^d^ pat(c.463+2T>C); ^e^ pat(c.769C>T)/mat(c.151_152dupGA); ^f^ pat(c.694C>T); ^g^ pat(c.769C>T)/mat(c.769C>T)

Supplementary Table 2. Identified variants of uncertain significance (VUS)

| **Patient** | **Referral** | **Gene** | **Transcript Change** | **Protein Change** | **Variant Type** | **ACMG/AMP criteria** | **Zygosity** | **ClinVar ID** |
| --- | --- | --- | --- | --- | --- | --- | --- | --- |
| P1 | Pediatric Emergency And Intensive Care Medicine | *KIF1A* | NM_0001244008.1: c.393C>A | p.Asn131Lys | class 3 | PM2, PP3 | heterozygote | 931381 |
| P2 | Neurology | *COL4A2* | NM_001846.2: c.1517G>A | p.Gly506Asp | class 3 | PM2, PP3 | heterozygote | 932136 |
| P3 | Cardiology | *LHCGR* | NM_000233.3: c.947+4_947+7del | - | class 3 | PM2, PP3, PP4 | heterozygote | 827819 |
| P4 | Gynecology | *PKD1* | NM_001009944.3: c.11872G>C | p.Ala3958Pro | class 3 | PM2, BP4 | heterozygote | 1027403 |
| P8 | Neurology | *GNAL* | NM_182978.3: c.394A>G | p.Lys132Glu | class 3 | PM2, PP2, PP3 | heterozygote | 3833066 |
| P9 | Pediatric Cardiology | *HCN4* | NM_005477.2: c.1492A>G | p.Ser498Gly | class 3 | PM2, PP3 | heterozygote | 4871888 |
| P10 | Neurology | *SPAST* | NM_014946.3: c.1315T>G | p.Phe439Val | class 3 | PM1, PM2, PP3 | heterozygote | 4871889 |
| P11 | Pediatric Neurology | *DNM1L/FAR1* | NM_001278464.1: c.1713+2T>A/ NM_032228.6: c.304A>G | -/p.Ile102Val | class 3/class3 | PM2/PM2, BP4 | heterozygote/homozygote | 2100336/ 1376691 |
| P12 | Pediatric Cardiology | *MYBPC3* | NM_000256.3: c.2441_2443delAGA | p.Lys814del | class 3 | **PS4**, BP3, BS3_SUP | heterozygote | 177700 |
| P13 | Pediatric Cardiology | *MYH7* | NM_000257.4: c.2481G>T | p.Trp827Cys | class 3 | **PS4**_SUP, PM2, PP3 | heterozygote | 1791933 |
| P14 | Pediatric Gastroenterology | *ABCB4* | NM_018849.3: c.833G>T | p.Arg278Met | class 3 | PM2, PP3 | heterozygote | 4846602 |
| P15 | Pediatric Cardiology | *CACNA1C* | NM_199460.3: c.2936T>C | p.Leu978Ser | class 3 | PM2, PP3 | heterozygote | 4871890 |
| P16 | Pediatric Emergency And Intensive Care Medicine | *DOK7* | NM_001301072.2:  c.532+4A>C | - | class 3 | PM2, PP3 | heterozygote | 2149114 |
| P17 | Neurology | *GNAO1* | NM_020988.3: c.529C>T | p.Arg177Ter | class 3 | PM2 | heterozygote | 1024118 |
| P18 | Pediatric Emergency And Intensive Care Medicine | *CUL7* | NM_014780.5: c.4886T>C | p.Leu1629Pro | class 3 | PM2, PP3 | heterozygote | 1465457 |
| P19 | Pediatric Oncology | *SDHB* | NM_003000.3:c .131T>G | p.Ile44Ser | class 3 | PM2, PP4, PP3 | heterozygote | 4871891 |
| P20 | Neurology | *PANK2* | NM_153638.3: c.1043A>G | p.Tyr348Cys | class 3 | PM2, PP4, PP3 | heterozygote | 4871892 |
| P21 | Neurology | *PRKCG* | NM_001316329.2: c.167T>A | p.Ile56Asn | class 3 | PM2, PP2, PP3 | heterozygote | 4871893 |
| P22 | Pediatric Cardiology | *FLNA* | NM_001110556.2: c.4240T>A | p.Tyr1414Asn | class 3 | PM2, PP3 | hemizygote^#^ | 4871894 |
| P23 | Pediatric Neurology | *HECW2* | NM_001348768.2: c.3909C>G | p.His1303Gln | class 3 | PM2, PP2, PP3 | heterozygote | 4871895 |
| P24 | Pediatric Neurology | *FLNA* | NM_001110556.2: c.4240T>A | p.Tyr1414Asn | class 3 | PM2, PP3 | hemizygote^#^ | 4871894 |
| P25 | Pediatric Neurology | *CLCN1* | NM_000083.3: c.1387T>G | p.Phe463Val | class 3 | PM2, PP3, PP4 | heterozygous | 4871897 |
| P26 | Pediatric Neurology | *HCN2* | NM_001194.4: c.1898T>G | p.Leu633Arg | class 3 | PM2, PM5, PP3 | heterozygous | 4871898 |
| P27 | Neonatology | *CFAP298* | NM_021254.4: c.829delA | p.Arg277fs | class 3 | PVS1_MOD, PM2, PP4 | homozygous | 1025340 |
| P28 | Pediatric Neurology | *HCN1* | NM_021072.4: c.728T>C | p.Met243Thr | class 3 | PM2, PP3, PM3 | heterozygous | 2027567 |
| P29 | Pediatric Neurology | *CHD2* | NM_001271.4: c.2708G>T | p.Arg903Ile | class 3 | PM2, PP3, PM5_SUP, PM1_SUP, PM1 | heterozygous | 1506347 |
| P30 | Pediatric Neurology | *GRIN2B* | NM_000834.4: c.1451G>T | p.Gly484Val | class 3 | PM2, PP2, PP3 | heterozygous | 4871899 |
| P31 | Pediatric Neurology | *NBEA* | NM_015678.4: c.2054T>C | p.Leu685Pro | class 3 | PM2, PP2, PP3 | heterozygous | 4871900 |
| P32 | Pediatric Neurology | *CYFIP2* | NM_001291722.2: c.679delC | p.Gln227fs | class 3 | PM2, PP3 | heterozygous | 4871901 |
| P33 | Pulmology | *TERT* | NM_198253.3: c.470C>A | p.Ala157Glu | class 3 | PM2, PP2, PP3 | heterozygous | 4871902 |
| P34 | Pediatric Neurology | *KIF2A* | NM_001098511.2: c.1369G>A | p.Asp457Asn | class 3 | PM2, PP3 | heterozygote^#^ | 4871903 |
| P35 | Pediatric Neurology | *KIF2A* | NM_001098511.2: c.1369G>A | p.Asp457Asn | class 3 | PM2, PP3 | heterozygote^#^ | 4871903 |
|  |  |  |  |  |  |  |  |  |
|  |  |  |  |  |  |  |  |  |
|  |  |  |  |  |  |  |  |  |

^#sibling^

**References**

1. Landrum MJ, Lee JM, Riley GR, Jang W, Rubinstein WS, Church DM, et al. ClinVar: public archive of relationships among sequence variation and human phenotype. Nucleic Acids Res. 2014 Jan;42(D1):D980–5. doi:10.1093/nar/gkt1113
